# Encapsulation of halocadmate anion via hydrogen bonding: synthesis and characterization of bis(diethylenetriamine)cobalt(III) complex containing hexabromocadmate anion

**DOI:** 10.55730/1300-0527.3500

**Published:** 2022-09-27

**Authors:** Vinit PRAKASH, Ritu BALA, Amrinder KAUR, Daron E. JANZEN

**Affiliations:** 1 Department of Chemistry, Maharishi Markandeshwar University, Sadopur, Haryana, India; 2 Department of Chemistry, UGC Centre for Advanced Studies-1, Guru Nanak Dev University, Punjab, India; 3 Department of Chemistry and Biochemistry, St. Catherine University, St. Paul, MN, USA

**Keywords:** Bromocadmate anions, encapsulation of anion, inorganic synthesis, second-sphere coordination, X-ray structure

## Abstract

In this work, the encapsulation of [CdBr_6_]^4−^ by six cations, [Co(dien)_2_]^3+^ has been described with the help of single crystal X-ray structural study in the complex, *mer*-[Co(dien)_2_]_2_[CdBr_6_]Br_2_. The complex anion, [CdBr_6_]^4−^ has been obtained through solution method while attempting to synthesize complex dianion, [CdBr_4_]^2−^. This newly synthesized complex has been initially characterized by elemental analyses and spectroscopic studies (IR, NMR and UV-Visible). IR and NMR studies have been used for the isomeric identification of [Co(dien)_2_]^3+^. Single crystal X-ray structure determination has revealed the presence of two complex cations, [Co(dien)_2_]^3+^, one complex anion, [CdBr_6_]^4−^, and two Br^−^ anions. The complex has crystallized in monoclinic crystal system with space group, P2_1_/n. The study of intermolecular interactions has confirmed the stability of crystal structure through N-H type H-bonding interactions besides electrostatic forces of attraction.

## 1. Introduction

The design, synthesis, and stabilization of a complex structure with predictable properties is always challenging task for chemists. However, the emergence of supramolecular networks of metal complexes has a deep effect to stabilize complex anions (like halocadmates) of different shapes, sizes, and functionalities [1]. Halocadmates(II) anions have been attracting significant consideration due to their varied geometric and polymeric frameworks ([Cd*_n_*X*_m_*]^(2^*^n^*^–^*^m^*^)^) like isolated molecules (0-D), infinite chains (1-D), two (2-D), and three dimensional lattices (3-D) [1–7]. In these cadmium(II) halides anions, cadmium to halide ratios range from 1:3 to 1:6. The shape of cadmium(II) halide complex anions are variable (like tetrahedral [8–9], complex chain structures [10–12], two-dimensional layered structures [13]) and these could be extracted/stabilized with the help of cations through noncovalent interactions (electrostatic interaction and H-bonding interactions). Recently, the role of topology and the H-bonding capabilities of a large counter ion, [Co(NH_3_)_6_]^3+^, [Co(en)_2_Cl_2_]^+^, [Co(phen)_3_]^3+^, have been shown in the isolation and stabilization of new anionic species, such as [CdBr_4_]^−^ [14], [CdI_4_]^2−^ [15],and [Cd_2_X_7_]^3−^ [16]. These studies indicate that compatibility in size and topology of complex cations are important to stabilize complex anions in crystal lattices through supramolecular networks. In this context, it becomes significant to probe the viability of isolating/stabilizing these anions as discrete entities. If the cations are endowed with proper functional groups that can effectively interact with one another to generate a “cage” and if the molecular topologies of the anions are such that a proper fitting is achieved, it may be possible to trap these anions.

In continuation to the previously reported literature, [Co(dien)_2_]^3+^ has been explored as a judicious choice because it has three grooved faces (for shape complementarity), it is large in size (size complementarity), and it has a three-unit positive charge (for electrostatic attraction). In addition, the periphery of the ion endowed with N–H groups (for H-bonding interactions) can show multiple N–H**···**X hydrogen bonds, which would lead to the lattice stabilization. The above mentioned considerations should favor the isolation/stabilization of [CdBr_6_]^4−^ using [Co(dien)_2_]^3+^ cations. Thus, this paper reports the successful synthesis and characterization of *mer*-[Co(dien)_2_]_2_[CdBr_6_]Br_2_. To the best of our knowledge, few reports are available on anion, [CdX_6_]^4−^ [17–22] having potential applications like [C_5_H_9_–NH_3_]_4_CdBr_6_ behaves as white-light emitting semiconductor having CIE chromaticity coordinates (0.33, 0.33), which are the same as that of the standard pure white-light emission and used for LED or flat-panel displays [23]. It also affects the luminescent properties of cations [18].

## 2. Materials and methods

### 2.1. General procedures and materials

Analytical grade reagents (from Merck) were used without any further purification. bis(diethylenetriamine)cobalt(III) chloride dihemihydrate has been prepared with the method reported in the literature [24, 25].

### 2.2. Synthesis of *mer*-bis(diethylenetriamine)cobalt(III) hexabromocadamate(II) bromide, (*mer*-[Co(dien)_2_]_2_ [CdBr_6_]Br_2_)

An aqueous solution (10 mL) of CdBr_2_ (0.3264 g, 0.0012 mol) and KBr (0.8568 g, 0.0072 mol) was added slowly in the aqueous solution (20 mL) of bis(diethylenetriamine)cobalt(III) chloride dihemihydrate (1.00 g, 0.0024 mol). The dark orange colored crystals that appeared after 2 h were collected by draining off the mother liquor and dried in air. The overall yield of the product was 77.1% (1.19 g) and it decomposed at 481 K. Solubility (25 **°**C ± 2) in water was 1.00 g/ 82 mL. Anal. Calc. (%) for *mer-*[Co(dien)_2_]_2_[CdBr_6_]Br_2_ (1282.23)= C: 14.99; H: 4.09; N: 13.11; Co: 9.19 and found C: 14.83; H: 4.04; N: 12.99; Co: 9.10, IR (cm^−1^) = ν_as_ 3196br (NH_2_); ν_s_ 3051m (NH_2_); ν 2883 m (CH_2_); δ 1559 m (NH_2_); δ 1475 m, 1459 m, (CH_2_-dien); **ω** 1324 m (CH_2_); ρ_r_ 1184 m (NH_2_); **ν** 1053 s (dien, C-N); **ρ**_r_ 924 w, 897 w, 866 w, 838 w (CH_2_-, NH_2_-, NH and CN); **ν** 518 (Co-N), UV-vis (solution): λ_max,_ nm (ɛ, M^−1^ cm^−1^) 466 (120); 342 (140), 224 (20040), NMR (D_2_O, δ (ppm)): ^1^H NMR: 4.68–4.62 (m, 10H, NH_2_ of dien), 3.20–3.30 (m, CH_2_ adjacent to NH_2_), 2.94–2.89 (m, CH_2_ adjacent to NH), ^13^C NMR: 51.02, 47.88, 46.58.

### 2.3. Instrumentation

Cobalt was determined by standard gravimetric method [26] and C, H, N were estimated microanalytically by FlashEA-1112 Series CHN-S analyzer. UV/Visible spectra were recorded using Shimadzu-1800 spectrophotometer in water as solvent. The cell holder of the spectrophotometer was thermostated at 25 °C (±1) for consistency in the recordings. The absorption spectrum was recorded between 800 and 200 nm. Infrared spectrum of the new complex was recorded using Varian Resolution Pro 660 FT/IR Spectrophotometer in KBr pellets. ^1^H and ^13^C-NMR spectra of new complexes were run in the solvent D_2_O at 25 °C (±1) by using AL-300 MZ JEOL 300MHz FT NMR spectrophotometer. The chemical shift values are expressed as δ value (ppm) downfield from tetramethylsilane as an internal standard reference. A NETZSCH STA449F1 instrument was used to carry out thermogravimetric analysis (TGA) in nitrogen atmosphere with a heating rate of 10 °C/min. The temperature ranges from 20 to 1000 °C.

### 2.4. Crystal structure determination

An orange prism crystal of C_16_H_52_Br_8_CdCo_2_N_12_having approximate dimensions of 0.48 × 0.19 × 0.15 mm was mounted on a MiTeGen micromount [27]. All measurements were made on a Rigaku XtaLAB mini diffractometer using graphite monochromated Mo-Kα radiation (λ = 0.71073 Å). The data were collected at −100°C using an Oxford Cryosystems desktop cooler [28]. The intensity data were corrected for absorption using CrysAlisPro [29]. Using Olex2 [30], the structure was solved with the SHELXT [31] structure solution program using Intrinsic Phasing and refined with the SHELXL [32] refinement. The crystal structure parameters are given in Table 1.

### 2.5. Structure solution and refinement for complex C_16_H_52_Br_8_CdCo_2_N_12_

All nonhydrogen atoms were refined anisotropically. All hydrogen atoms were refined using the riding model. Though diffraction intensities and overall quality of the data was high, attempts to refine the hydrogen atoms coordinates for the hydrogens bonded to the nitrogen atoms yielded unreasonable N-H distances. The final cycle of full-matrix least-squares refinement on F^2^ (based on 3189 observed reflections (R_int_ = 0.0192) and 178 variable parameters and converged (largest parameter shift was 0.00 times its esd) with unweighted and weighted agreement factors of: R_1_ = 0.0403, wR_2_ = 0.1139.

## 3. Results and discussion

### 3.1. Synthesis

The reaction between isomeric mixture (*s*-*fac*:*u*-*fac*:*mer* is 7:28:65) of [Co(dien)_2_]Cl_3_, CdBr_2_, and KBr was carried in 2:3:6 molar ratio in order to obtain [Co(dien)_2_]_2_[CdBr_4_]_3_ in aqueous medium (see Eq. (i)). However, the attempt to obtain the product according to equation (i) was unsuccessful. The elemental analysis of the product obtained from equation (i) corresponds to formula [Co(dien)_2_]_2_[CdBr_6_]Br_2_ instead of [Co(dien)_2_]_2_[CdBr_4_]_3._ Therefore, reaction was repeated again by changing the molar ratios of reactant as given in equation (ii).


(i) $$2[\text{Co}{\left(\text{dien}\right)}_{2}]{\text{Cl}}_{3}+3{\text{CdBr}}_{2}+6\text{KBr}\to {\left[\text{Co}{\left(\text{dien}\right)}_{2}\right]}_{2}{\left[{\text{CdBr}}_{4}\right]}_{3}+6\text{KCl},$$

(ii) $$2[\text{Co}{\left(\text{dien}\right)}_{2}]{\text{Cl}}_{3}+{\text{CdBr}}_{2}+6\text{KBr}\to {\left[\text{Co}{\left(\text{dien}\right)}_{2}\right]}_{2}[{\text{CdBr}}_{6}]{\text{Br}}_{2}+6\text{KCl}.$$

The composition of both the products obtained according to reactions given in equations (i) and (ii) was identical, i.e. [Co(dien)_2_]_2_[CdBr_6_]Br_2_ as indicated initially by the elemental analysis. On the other hand, the complex was formed with meridonial (*mer*) isomer. Moreover, yield of the *mer* has been improved due to intramolecular conversion of *s-fac or u-fac isomer* of [Co(dien)_2_]^3+^ to *mer* isomer through (i) intramolecular twist and (ii) bond rupturing in aqueous medium [33].

### 3.2. Infrared spectroscopy

The infrared spectrum (see Fig S1) of [Co(dien)_2_]^3+^ is much more distinctive and are useful for the identification of its isomers [34]. The most useful regions for their characterization are 950–800 cm^−1^ and 3000–2800 cm^−1^. In case of *mer*-isomer, the former region exhibits band of quartet (which is assigned for CH_2_-, NH_2_-, and NH-rocking modes and CN skeletal vibrations) and other two isomers (*s-* and *u-fac*) exhibit either fewer peaks or broad absorptions. In the latter region (for CH_2_ stretching vibrations), very weak absorption band was reported for *fac*- isomers but strong for *mer* [34]. The CH_2_ stretching vibrations have lower intensities as compared to the NH_2_ stretching modes and are hardly recognizable for *fac-* isomers.

In IR spectrum of complex shows a band of quartet in the region 950–800 cm^−1^ indicating the presence of *mer* isomer. Moreover, the region 3000–2800 cm^−1^ (cationic CH_2_ stretching vibrations) appears to be strong with number of bands in case of complex. From the interpretation of IR spectrum it is concluded that the complex contains *mer*- isomer. The IR band assignments of new complex was compared with already reported [Co(dien)_2_]Cl_3_ in the literature [34]. The NH_2_ bending vibrations were observed at 1559 cm^−1^ for newly synthesized complex. However, for [Co(dien)_2_]Cl_3_, the NH_2_ bending vibrations appeared at 1572 cm^−1^ [24, 34]. The average 13 cm^−l^ lowering in this frequency may indicate the weakening of N-H bonds due to presence of strong H-bonding interactions between cation and halocadmate anion.

### 3.3. NMR spectroscopy

In ^1^H NMR spectra of *s-fac* or *mer-*[Co(dien)_2_]^3+^ isomers, there is presence of only two nonequivalent methylene groups due to which only quartet (for CH_2_ adjacent to NH_2_) and quintet (for CH_2_ adjacent to NH) were expected [34]. In case of *u-fac* isomer, complex splitting pattern would be expected because of four nonequivalent methylene groups. However, in title complex, two multiplets (for CH_2_ adjacent to NH_2_and NH) were observed in the range 3.20–3.30 ppm and 2.94–2.89 ppm, respectively (Figure S3) indicating the formation of *s-fac* or *mer-*[Co(dien)_2_]^3+^ isomers. Moreover, for NH_2_ of dien, instead of a singlet (at 4.70 ppm), a complex pattern was observed (δ in the range 4.68–4.62) which also supports the presence of isomer *mer* and *u-fac*-isomers. Therefore, ^1^H NMR is not very much supporting to identify the particular isomer.

Furthermore, the ^13^C NMR pattern appears to be completely diagnostic for the identification of isomers. The ^13^C NMR pattern of complex shows three δ values for methylene group adjacent to NH and NH_2_ (δ value at 51.11, 47.94, and 46.63 ppm) indicating the formation of *mer* isomer. ^1^H and ^13^C NMR chemical shift values for newly synthesized complex is in good agreement with the literature and helps in the isomeric confirmation of [Co(dien)_2_]^3+^ [33–35].

### 3.4. UV–visible titration studies

The UV–visible spectra of the three isomers (*s-fac*, *u-fac*, and *mer*) containing [Co(dien)_2_]^3+^ were reported in the literature [25]. Mainly three transitions, two d-d (^1^A_1g_ →^1^T_1g_ and ^1^A_1g_ →^1^T_2g_) and one charge transfer band (N(σ) →e_g_(σ*) were reported. For the complex (Figure S2), the absorption maxima observed at 466, 342, and 224 for ^1^A_1g_ →^1^T_1g,_^1^A_1g_ →^1^T_2g_ transitions and N(σ) →e_g_(σ*) charge transfer band, respectively. As all the absorption maxima (λ_max_) were observed for cation, [Co(dien)_2_]^3+^ were near the configuration of isomer *mer-*[Co(dien)_2_]^3+^.

### 3.5. X-ray crystallography

**Figure 1:**
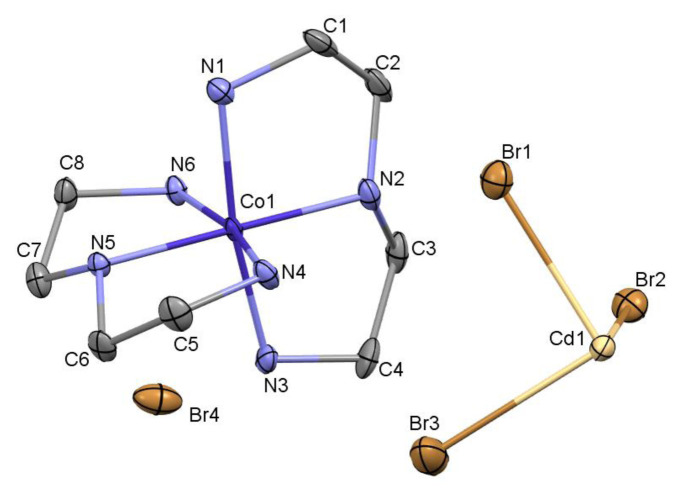
Anisotropic thermal ellipsoid plot of the asymmetric unit of *mer*-[Co(dien)_2_]_2_[CdBr_6_]Br_2_ (50% probability ellipsoids). Hydrogen atoms were omitted forclarity.

**Figure 2:**
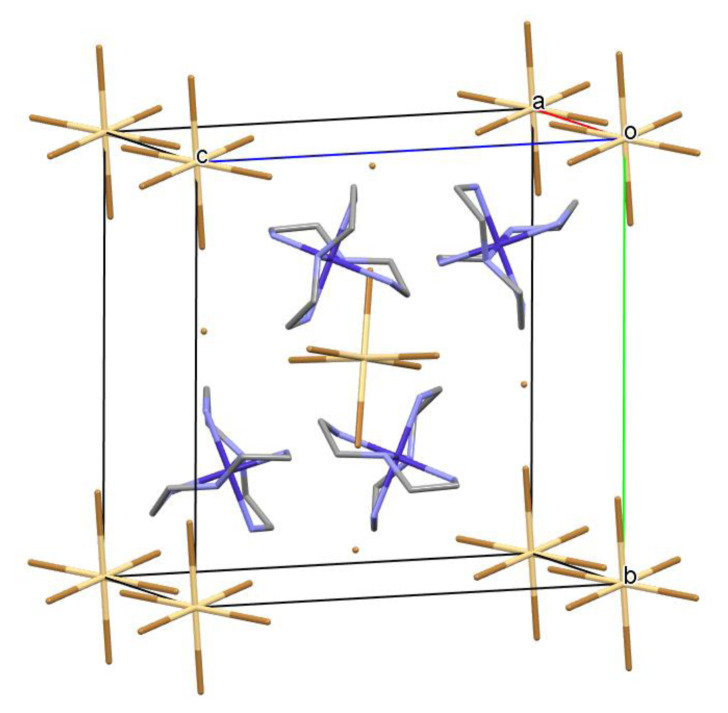
Unit cell packing diagram of *mer*-[Co(dien)_2_]_2_[CdBr_6_]Br_2_. Hydrogen atoms were omitted for clarity.

The asymmetric unit of complex consists of one complete cation, [Co(dien)_2_]^3+^, one bromide anion, and one half of a [CdBr_6_]^4−^ anion located on an inversion center. The formula of the complex may be considered to include two [Co(dien)_2_]^3+^ cations, one complete [CdBr_6_]^4−^ anion, and two bromide anions. The cobalt center is roughly octahedral with two dien ligands bonded in meridional coordination geometry. Anisotropic thermal ellipsoid plot of the asymmetric unit of complex is given in Figure 1 and unit cell packing diagram in Figure 2. Co-N bonds fall within typical values. The cobalt bonds to the terminal N atoms of the dien units (Co1-N1 1.952(6) Å, Co1-N3 1.950(5) Å, Co1-N4 1.956(5) Å, Co1-N6 1.940(6) Å) are slightly longer than the Co-N bonds to the secondary nitrogen (Co1-N2 1.924(6) Å, Co1-N5 1.917(5) Å)(Table 2). Moreover, N-Co-N angles show small distortions from ideal angles. This is likely due to the steric constraints of the ethylene straps of the dien ligand for cobalt coordination to the 1,4 and 4,7 nitrogen positions. Each dien ligand adopts a conformation closer to mirror symmetry than to two-fold rotational symmetry. Neither dien ligand is subject to any actual crystallographic symmetry.

The rare [CdBr_6_]^4−^ anion was an unsuspected surprise in this structure. The geometry around the Cd is roughly octahedral with average bond angle *cis*-Br-Cd-Br 90°(2) (with a narrow range (87.35(2) – 92.65(2)°) (Table 3) and *trans-*Br-Cd-Br is 180°. Three unique Cd-Br bond lengths are present with a large range (Cd1-Br1 2.6537(8) Å, Cd1-Br2 2.7545(7) Å, Cd1-Br3 2.856(8) Å). There are very few other structures with discrete [CdBr_6_]^4−^ anions [17–22]. Out of these, the most closely related is cobalt coordination compound, bis(tris(ethylenediamine)cobalt(III)) hexabromocadmium(II) dibromide dihydrate [18]. In [Co(en)_3_]_2_[CdBr_6_].Br_2_.2H_2_O, the discrete anion, [CdBr_6_]^4−^contains average bond length 2.788(1) Å (ranging from 2.686 (1) to 2.889(1) Å) and average bond angle *cis*-Br-Cd-Br is 90 (4)° (ranging from 85.57 (3) to 94.13 (3)°) and *trans*-Br-Cd-Br 180°, respectively.

**Figure 3:**
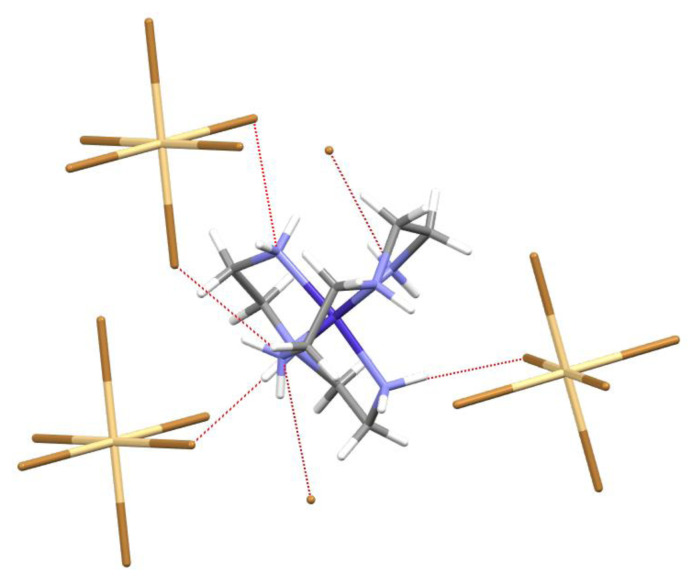
Partial packing diagram of *mer*-[Co(dien)_2_]_2_[CdBr_6_]Br_2_. Each [Co(dien)_3_]^3+^ cation shows hydrogen bonding with 3 unique [CdBr_6_]^4−^ anions and 2 unique bromide anions.

**Figure 4:**
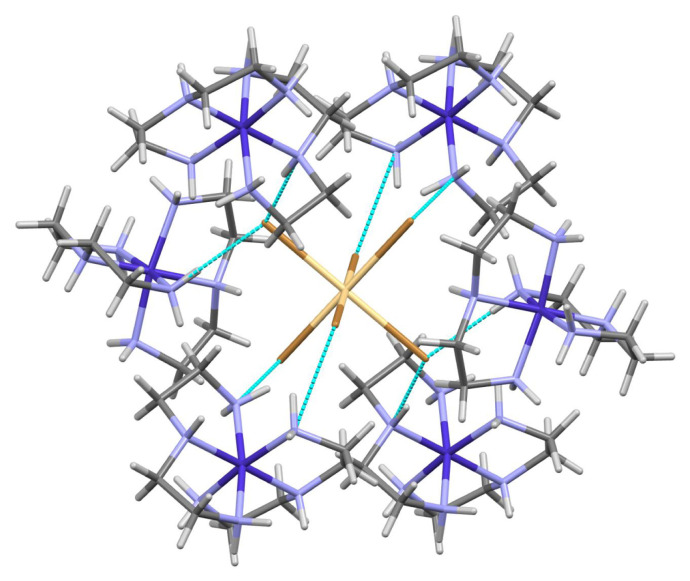
Partial packing diagram of *mer*-[Co(dien)_2_]_2_[CdBr_6_]Br_2_. Each [CdBr_6_]^4−^ anion shows hydrogen bonding with six [Co(dien)_2_]^3+^ cations.

Extensive hydrogen bonding interactions are present. Details of the hydrogen bonding are found in Table 4. As the nitrogen hydrogens were modeled in ideal positions, the donor–acceptor distances and overall motifs will be the focus of this analysis. For the purposes of this analysis, no nonclassical hydrogen bonding is considered (such as C-H bonds as hydrogen bond donors) and hydrogen bonds are described only for D-H…A angles of >140° and bromine donor nitrogen…acceptor bromine distances are described. Each bromine atom of the anion, [CdBr_6_]^4−^ act as hydrogen bond acceptors. Atom Br2 acts as an acceptor for a pair of hydrogen bonds with N-H groups of the same cation (Br2…H1A-N1 = 3.325(5) Å, Br2…H4A-N4 = 3.406(6) Å). Atom Br1 acts as an acceptor for a hydrogen bond with an N-H groups of a different cation (Br1…H2-N2 = 3.534(6)Å ). Atom Br3 acts as an acceptor for a pair of hydrogen bonds with N-H groups from two different cations (Br3…H6A-N6 =3.259(6) Å, Br3…H5-N5 =3.232(5) Å). The [CdBr_6_]^4−^ anion lies on an inversion center and these interactions are duplicated so that each bromine atom of the anion acts as an acceptor (Figure 3). Each anion, [CdBr_6_]^4−^ undergoes hydrogen bond acceptor interactions with a total of six [Co(dien)_2_]^3+^ cations (Figure 4) and thus facilitates the encapsulation of the anion, [CdBr_6_]^4−^. The unligated (ionic) bromide also acts as a hydrogen bond acceptor (Figure 3). The atom Br4 acts as an acceptor for two pairs of pairwise interactions from different N-H groups of different cations. One set of pairwise acceptor interactions includes Br4…H3B-N3 (3.338(6) Å ) and Br4 …H4B-N4 (3.434(6) Å). The second set of pairwise interactions includes Br4…H1B-N1 (3.351(5) Å) and Br4 …H6B-N6 (distance = 3.619(5) Å). Each bromide anion thus undergoes hydrogen bond acceptor interactions with 2 unique [Co(dien)_2_]^3+^ cations. The *mer*-[Co(dien)_2_]^3+^ cation engages in extensive hydrogen bonding in this structure as a hydrogen bond donor. Each *mer*-[Co(dien)_2_]^3+^ cation acts as a hydrogen bond donor to two unique [CdBr_6_]^4−^ anions and two unique bromide anions.

A unit cell diagram shows that [CdBr_6_]^4−^ anions pack at the unit cell corners and body center, with [Co(dien)_2_]^3+^ cations and bromide anions packing in the interstices (Figure 2). The packing motif of this structure indicates that there is not a simple obvious vector preference for the hydrogen-bonding pattern, as the [CdBr_6_]^4−^ anions and unligated anionic bromides are both involved extensively as hydrogen-bonding acceptors. No unit cell axes or other directions show large differences in strength and number of hydrogen bonding interactions from others.

### 3.6. Thermal gravimetric analysis (TGA)

**Figure 5:**
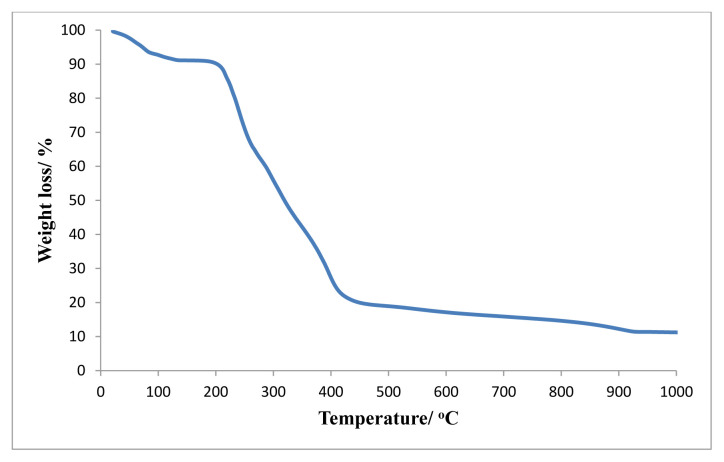
TGA of *mer*-[Co(dien)_2_]_2_[CdBr_6_]Br_2_.

Thermal stability of *mer*-[Co(dien)_2_]_2_[CdBr_6_]Br_2_ was determined in the temperature range 20 to 1000 °C under nitrogen flow (Figure 5). The first weight loss was observed in the range of 20 to 100 °C due to release of moisture (0.559 mg, 6.98%). The complex remains stable up to 210 °C. The decomposition of complex occurs at 210 °C. This step involves the continuous weight loss indicating the removal of en and 8Br^−^ in the temperature range of 210–430 °C. The experimental weight loss (5.74 mg, 77.10%) is consistent with the theoretical weight loss (6.10 mg, 82.04%). After 430 °C, no weight loss was observed which delineates the residues of Cd and Co.

## 4. Conclusion

This article investigates the stabilization of complex anion, [CdBr_6_]^4−^ using complex cation, [Co(dien)_2_]^3+^ through H-bonding interactions besides electrostatic forces of attraction. Each anion, [CdBr_6_]^4−^ undergoes hydrogen bond acceptor interactions with a total of six unique cations, [Co(dien)_2_]^3+^ which behave as H-bond donors and this interaction facilitates the encapsulation of the anion. The complex cation was identified as *mer* isomer with the help of UV–visible, IR, and NMR spectroscopy which was also supported by single crystal X-ray diffraction analysis.

## Supplementary information

Encapsulation of halocadmateanion via hydrogen bonding: synthesis and characterization of bis(diethylenetriamine)cobalt(III) complex containing hexabromocadmate anion

### Table of contents

**Table:** 

Page no.	Figures	Content
1	Figures S1	UV-visible spectra of (A) [Co(dien)_2_]Cl_3_ and *mer-*[Co(dien)_2_]_2_[CdBr_6_]Br_2_ (B) Expanded area of region 250 to 600 nm.
2	Figures S2	IR spectrum of *mer-*[Co(dien)_2_]_2_[CdBr_6_]Br_2_
3	Figures S3	^1^HNMR spectrum of *mer-*[Co(dien)_2_]_2_[CdBr_6_]Br_2_

Figure S1 UV–visible spectra of (A) [Co(dien)_2_]Cl_3_ and *mer-*[Co(dien)_2_]_2_[CdBr_6_]Br_2_ (B) Expanded area of region 250 to 600 nm.

Figure S2 IR spectrum of *mer-*[Co(dien)_2_]_2_[CdBr_6_]Br_2_.

Figure S3 (a) ^1^HNMR of *mer-*[Co(dien)_2_]_2_[CdBr_6_]Br_2_, (b) magnified region from 2.65 to 3.45 ppm.

## Figures and Tables

**Table 1:** Crystal data and structure refinement of *mer*-[Co(dien)_2_]_2_ [CdBr_6_]Br_2_.

Empirical formula	C_16_H_52_Br_8_CdCo_2_N_12_
M_r_(g mol^−1^)	1282.23
Crystal system	monoclinic
Space group	P2_1/n_
*a* (Å)	8.8599(3)
*b* (Å)	13.5471(4)
*c* (Å)	14.7561(5)
β (°)	100.118(3)
*V* (Å^3^)	1743.57(10)
*Z*	2
*D_x_*(g cm^−3^)	2.3890
μ (cm^−1^)	107.43
*R*_1_ (I > 2.00 σ(I))	0.0403
*wR*_2_ (all reflections)	0.1139
Goodness of fit	1.045

**Table 2:** Bond lengths (Å) of *mer*-[Co(dien)_2_]_2_[CdBr_6_]Br_2_ (Å).

Cd1	Br2	2.7545(7)
Cd1	Br2^1^	2.7545(7)
Cd1	Br1	2.6537(8)
Cd1	Br1^1^	2.6537(8)
Cd1	Br3	2.7856(8)
Cd1	Br3^1^	2.7856(8)
Co1	N5	1.917(5)
Co1	N6	1.940(6)
Co1	N3	1.950(5)
Co1	N4	1.956(5)
Co1	N1	1.942(6)
Co1	N2	1.924(6)
N5	C7	1.453(8)
N5	C6	1.464(8)
N6	C8	1.480(8)
N3	C4	1.479(8)
N4	C5	1.481(8)
N1	C1	1.471(9)
N2	C3	1.470(9)
N2	C2	1.463(9)
C8	C7	1.488(10)
C1	C2	1.480(11)
C4	C3	1.493(10)
C5	C6	1.483(9)

Symmetry operators:(1) -2-X,1-Y,1-Z.

**Table 3:** Bond angles (°) of *mer*-[Co(dien)_2_]_2_[CdBr_6_]Br_2_.

Br2^1^	Cd1	Br2	180.0	N1	Co1	N3	170.1(2)
Br2^1^	Cd1	Br3^1^	89.25(2)	N1	Co1	N4	89.4(2)
Br2	Cd1	Br3^1^	90.76(2)	N2	Co1	N6	95.0(2)
Br2	Cd1	Br3	89.24(2)	N2	Co1	N3	84.7(2)
Br2^1^	Cd1	Br3	90.75(2)	N2	Co1	N4	95.3(2)
Br1	Cd1	Br2^1^	87.35(2)	N2	Co1	N1	85.7(2)
Br1^1^	Cd1	Br2^1^	92.65(2)	C7	N5	Co1	108.8(4)
Br1^1^	Cd1	Br2	87.36(2)	C7	N5	C6	117.8(5)
Br1	Cd1	Br2	92.64(2)	C6	N5	Co1	109.9(4)
Br1	Cd1	Br1^1^	180.0	C8	N6	Co1	111.0(4)
Br1	Cd1	Br3^1^	89.56(2)	C4	N3	Co1	110.8(4)
Br1	Cd1	Br3	90.44(2)	C5	N4	Co1	108.3(4)
Br1^1^	Cd1	Br3	89.56(2)	C1	N1	Co1	109.1(4)
Br1^1^	Cd1	Br3^1^	90.44(2)	C3	N2	Co1	107.8(4)
Br3	Cd1	Br3^1^	180.0	C2	N2	Co1	109.6(4)
N5	Co1	N6	84.5(2)	C2	N2	C3	116.1(6)
N5	Co1	N3	94.9(2)	N6	C8	C7	108.5(5)
N5	Co1	N4	85.2(2)	N1	C1	C2	108.9(6)
N5	Co1	N1	94.7(2)	N3	C4	C3	109.1(6)
N5	Co1	N2	179.3(2)	N4	C5	C6	107.9(5)
N6	Co1	N3	91.9(2)	N2	C3	C4	105.8(5)
N6	Co1	N4	169.6(2)	N5	C7	C8	105.8(5)
N6	Co1	N1	91.6(2)	N5	C6	C5	104.4(5)
N3	Co1	N4	88.8(2)	N2	C2	C1	106.1(5)

Symmetry operators: (1) 2-X,1-Y,1-Z.

**Table 4:** Hydrogen bonds of *mer*-[Co(dien)_2_]_2_[CdBr_6_]Br_2_.

Donor	H	Acceptor	d(D...A)/Å	d(D-H)/Å	d(H...A)/Å	D-H...A/°
N1	H1A	Br2^1^	3.325(5)	0.91	2.56	142.5
N4	H4A	Br2^1^	3.406(6)	0.91	2.58	151.0
N2	H2	Br1^2^	3.534(6)	1.00	2.64	149.1
N1	H1B	Br4^3^	3.351(5)	0.91	2.56	145.2
N3	H3B	Br4	3.338(6)	0.91	2.49	155.5
N4	H4B	Br4	3.434(6)	0.91	2.64	146.0
N5	H5	Br3	3.232(5)	1.00	2.25	168.5
N6	H6A	Br3	3.259(6)	0.91	2.35	173.1
N6	H6B	Br4^3^	3.619(5)	0.91	2.85	143.6

Symmetry operators:(1) 1-X,1-Y,1-z (2) 1.5-X,0.5+Y,1.5-Z (3) 0.5+X,1.5-Y,0.5+Z.
