# Supplementary figures and images for: Encapsulation of halocadmate anion via hydrogen bonding: synthesis and characterization of bis(diethylenetriamine)cobalt(III) complex containing hexabromocadmate anion
Source: Turk J Chem. 2022 Sep 27;46(6):2036–45. doi: 10.55730/1300-0527.3500 (PMC10446937; doi:10.55730/1300-0527.3500)

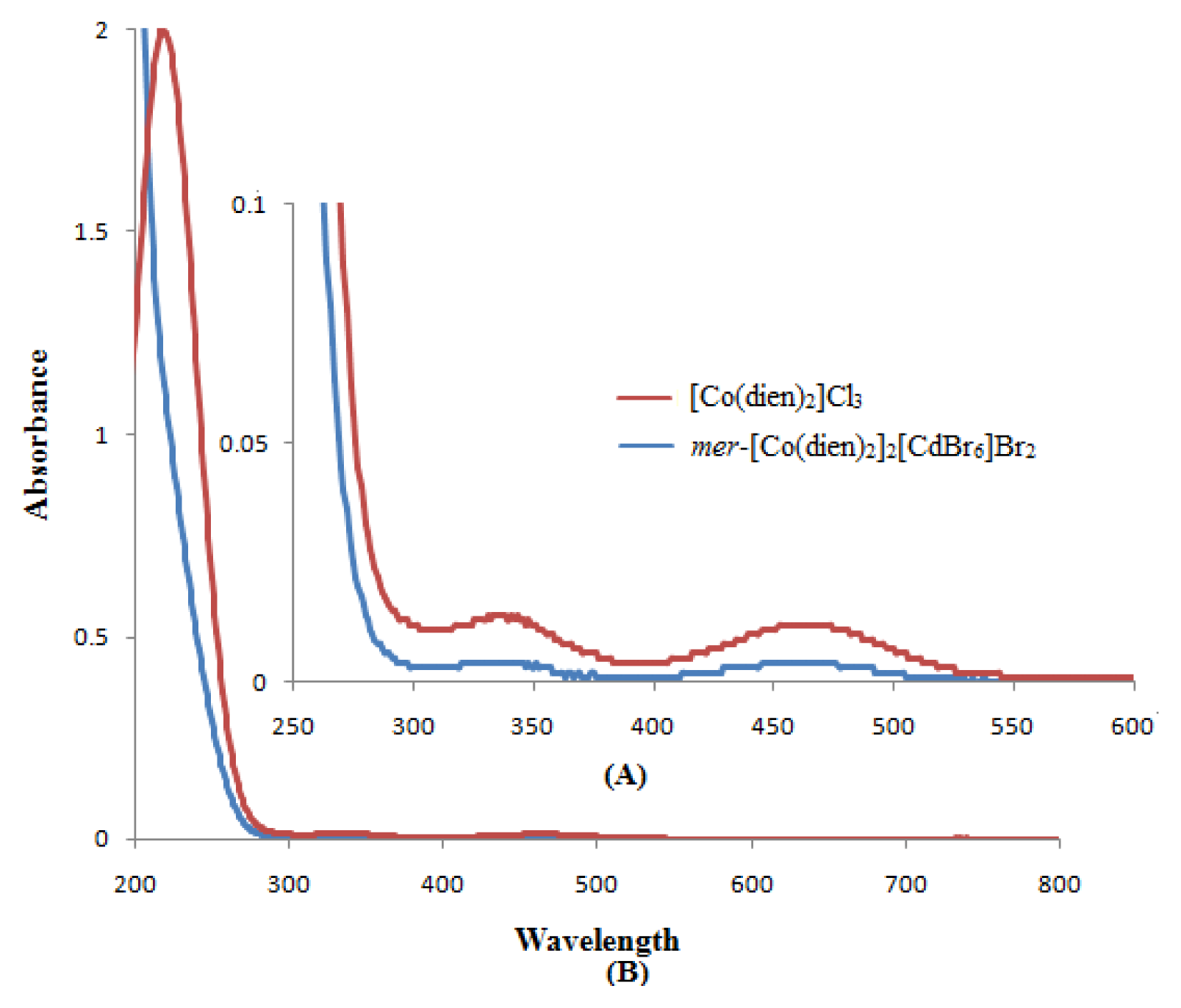

Supplement: Figure S1 — UV–visible spectra of (A) [Co(dien)2]Cl3 and mer-[Co(dien)2]2[CdBr6]Br2 (B) Expanded area of region 250 to 600 nm. [file turkjchem-46-6-2036s1.tif]

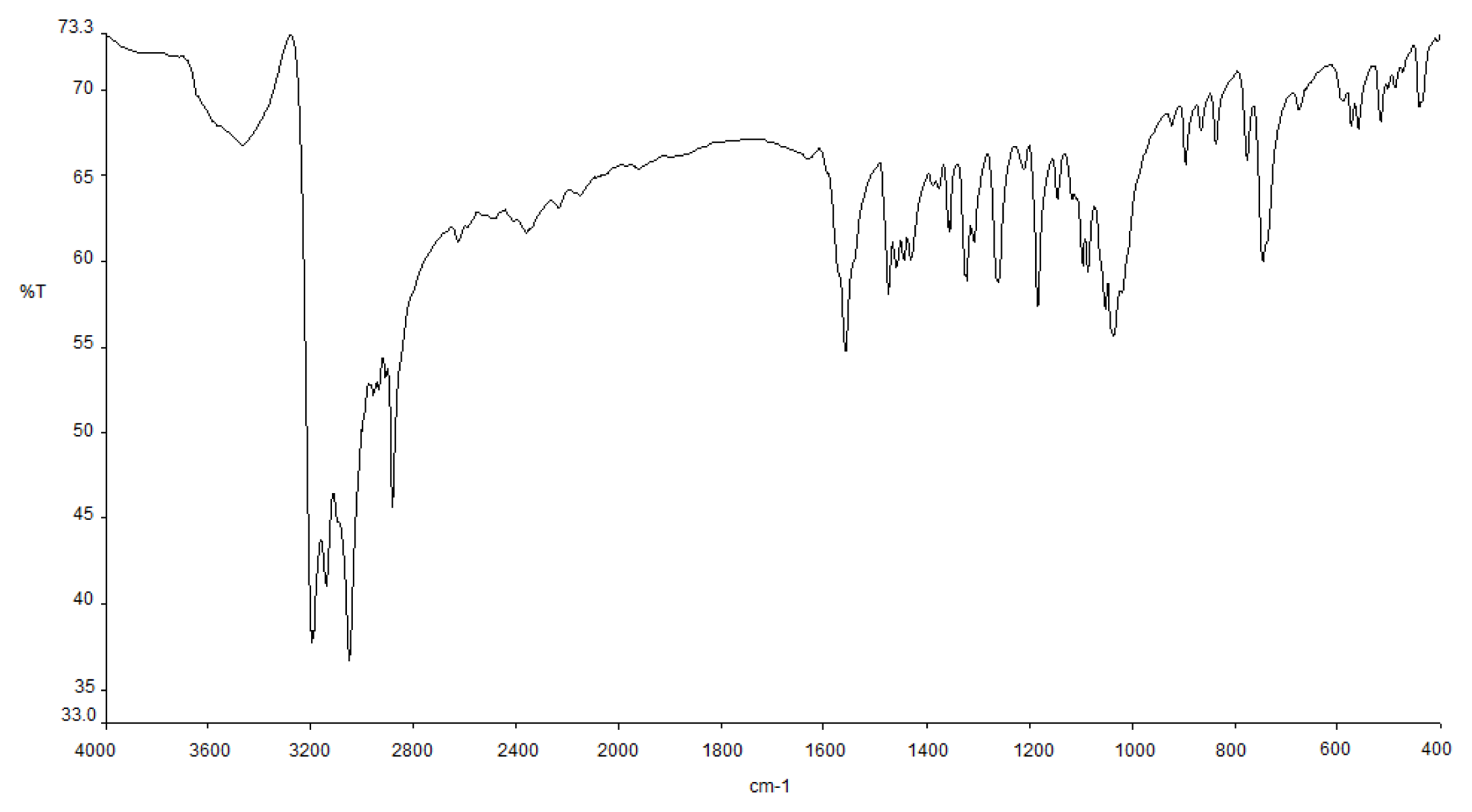

Supplement: Figure S2 — IR spectrum of mer-[Co(dien)2]2[CdBr6]Br2. [file turkjchem-46-6-2036s2.tif]

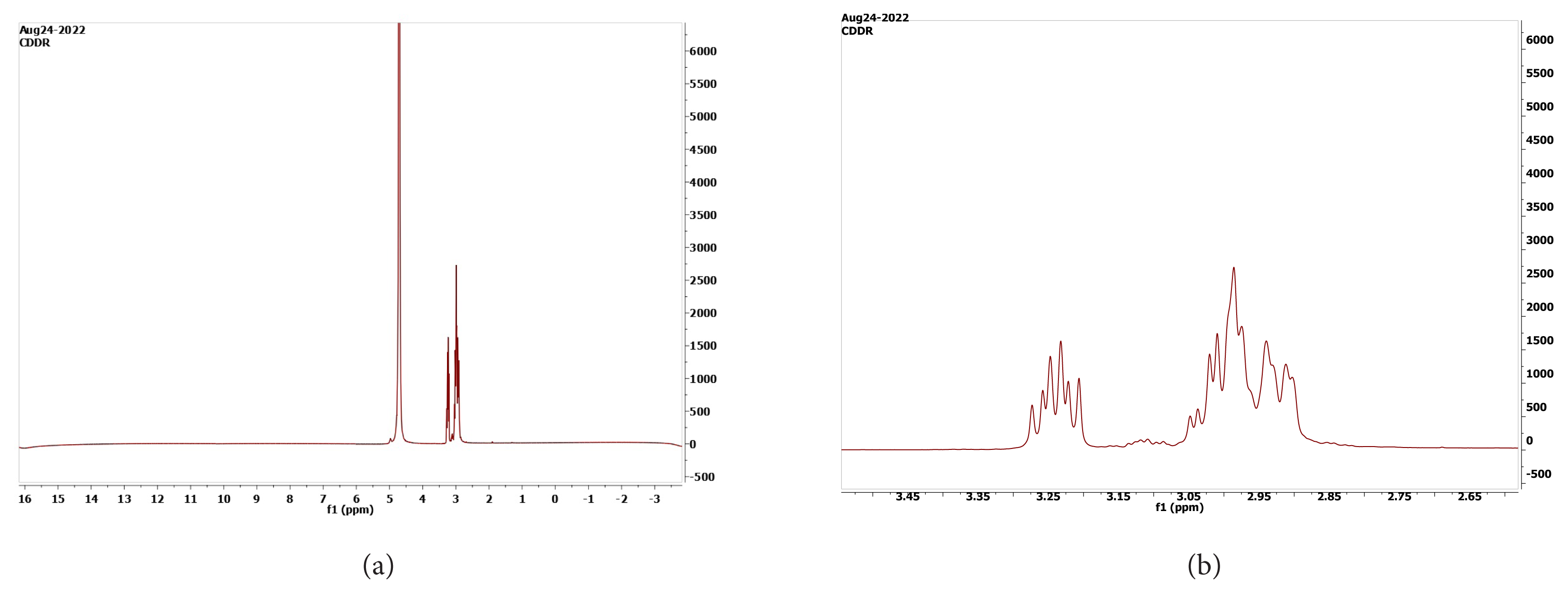

Supplement: Figure S3 — (a) 1HNMR of mer-[Co(dien)2]2[CdBr6]Br2, (b) magnified region from 2.65 to 3.45 ppm. [file turkjchem-46-6-2036s3.tif]
